# Reproducible vasovagal syncope triggered by indwelling pleural catheter drainage: A case of vagal hypersensitivity

**DOI:** 10.1016/j.rmcr.2026.102529

**Published:** 2026-10-29

**Authors:** Parita Shah, Lennon Colin, Irvin Lien, Pushan Jani

**Affiliations:** a GMERS Medical College, Gotri, Vadodara, Gujarat, India; b McGovern Medical School at UTHealth, Houston, TX, USA; c Division of Pulmonary and Critical Care Medicine, McGovern Medical School, Houston, TX, USA

**Keywords:** Indwelling pleural catheter, Vasovagal syncope, Vagus nerve, Lung adenocarcinoma, PleurX, Malignant pleural effusions

## Abstract

Indwelling pleural catheters (IPCs) are generally safe and effective for symptomatic relief and drainage of malignant pleural effusions. Common complications include infection and blockage; however, repeated vasovagal syncopal symptoms during drainage are rare. We report the case of a 62-year-old male with metastatic lung adenocarcinoma complicated by recurrent malignant pleural effusions, who experienced repeated near-syncopal episodes with profound bradycardia (HR < 30 bpm) during the first thoracentesis, during IPC placement and recurrent vasovagal symptoms during subsequent drainage episodes of the catheter at home for two more times. Symptoms were reproducible, occurring even with small-volume removals (<300mL). These episodes were theorized to be due to direct and indirect stimulation of the vagal nerve. The patient was also on atenolol (a betablocker), meaning that his compensatory sympathetic response may have been blunted. Management focused on discontinuation of atenolol and modification of IPC drainage technique (supine positioning and slower flow), which mitigated the episodes. Clinicians must recognize that vagal hypersensitivity, near syncope, and hemodynamic instability are possible complications of IPC drainage.

## 1 Introduction

Malignant pleural effusions (MPEs) are common and affect 150,000 patients annually in the United States [1]^.^ Current guidelines recommend indwelling pleural catheters (IPCs) for outpatient management of recurrent malignant and benign pleural effusions [2]. Generally, IPCs are considered safe, with well-described complications including infection, blockage, and pleural loculation [3], [4], [5]. Vasovagal reactions have been described during invasive pleural interventions, occurring in up to 10% of standard procedures [6], with the inciting stimuli being primarily pain or needle insertion. However, recurrent and reproducible vasovagal episodes triggered specifically by IPC drainage—especially at low volumes—are not well characterized.

## 2 Case presentation

This is a 62-year-old man with past medical history of hyperthyroidism and hypertension who presented to the Emergency Department (ED) for progressive dyspnea on exertion and was found to have a large right-sided pleural effusion. Initial cardiac evaluation, including electrocardiography and biomarkers, was unremarkable. A right-sided thoracentesis yielded 600mL of exudative fluid, during which the patient experienced a transient vasovagal episode (HR30s bpm) that resolved spontaneously after 30 seconds. Pleural fluid cytology confirmed metastatic lung adenocarcinoma, and the patient was discharged with follow up with oncology and pulmonology.

Two weeks after his initial presentation, the patient was evaluated by pulmonology. He had worsening dyspnea and a recurrent large right-sided pleural effusion. Consequently, he was scheduled for an IPC placement for symptomatic relief. Again, following the placement of the IPC and drainage of approximately 1L of pleural fluid, the patient experienced profound bradycardia (HR 20s bpm) accompanied by nausea, requiring 0.3 mg of atropine with recovery of heart rate over 5 minutes. The following day at home, the patient drained another 300 mL of pleural fluid via the IPC. He experienced another episode of near-syncope, prompting evaluation at the ED where he was again found to be hypotensive. A cardiac workup including troponin, ECG, and echocardiogram was unremarkable. The consistent timing and resolution of symptoms with the cessation of drainage suggested a procedure-related cause rather than a primary cardiac etiology.

On a subsequent pulmonary visit, vitals were monitored constantly, and drainage was performed slowly in the supine position. Additionally, the patient withheld his atenolol in the morning. The procedure was stopped the moment the patient felt symptoms of discomfort at 225 mL drained. Notably, the patient experienced no lightheadedness or bradycardia during this controlled session. One week later, in the pulmonary clinic, the patient tolerated 550 mL of drainage without recurrence of symptoms with similar supine positioning, slow drainage, and a reduced atenolol dosage. Post drainage chest radiography and chest ultrasound demonstrated complete re-expansion of the lung without evidence of residual pleural fluid or a trapped/nonexpandable lung. The pleural fluid output gradually decreased over time, and the PleurX catheter was removed approximately two months after its initial placement. Intrapleural pressure was not formally measured with manometry during drainage because there was low clinical suspicion for a trapped lung.

## 3 Discussion

The mechanisms behind these near-syncopal episodes are likely multifactorial. Rapid pleural fluid removal alters intrathoracic pressure and stimulates vagal afferent fibers within the pulmonary plexus, which innervates the visceral pleura [7]. Sudden lung re-expansion and pleural stretch activates mechanoreceptors, triggering a reflex arc via the nucleus tractus solitarius that increases parasympathetic output and suppresses sinoatrial node activity, resulting in bradycardia and hypotension. This pathway mirrors the Bezold-Jarisch reflex in which mechanical stimulation of cardiopulmonary receptors yields vagal efferent hypersensitivity [8].

Additionally, acute reductions in pleural pressure may transiently decrease venous return and left ventricular preload, similar to a Valsalva maneuver [9], further augmenting reflex vagal tone [10](Fig. 1). Although drainage-related excessively negative intrapleural pressure can occur in the setting of a trapped lung or nonexpandable lung, this was considered unlikely in our patient. Post-drainage chest radiography and chest ultrasound demonstrated complete lung re-expansion without evidence of residual pleural fluid or a trapped lung. Intrapleural pressure was not directly measured with manometry.

**Fig. 1:**
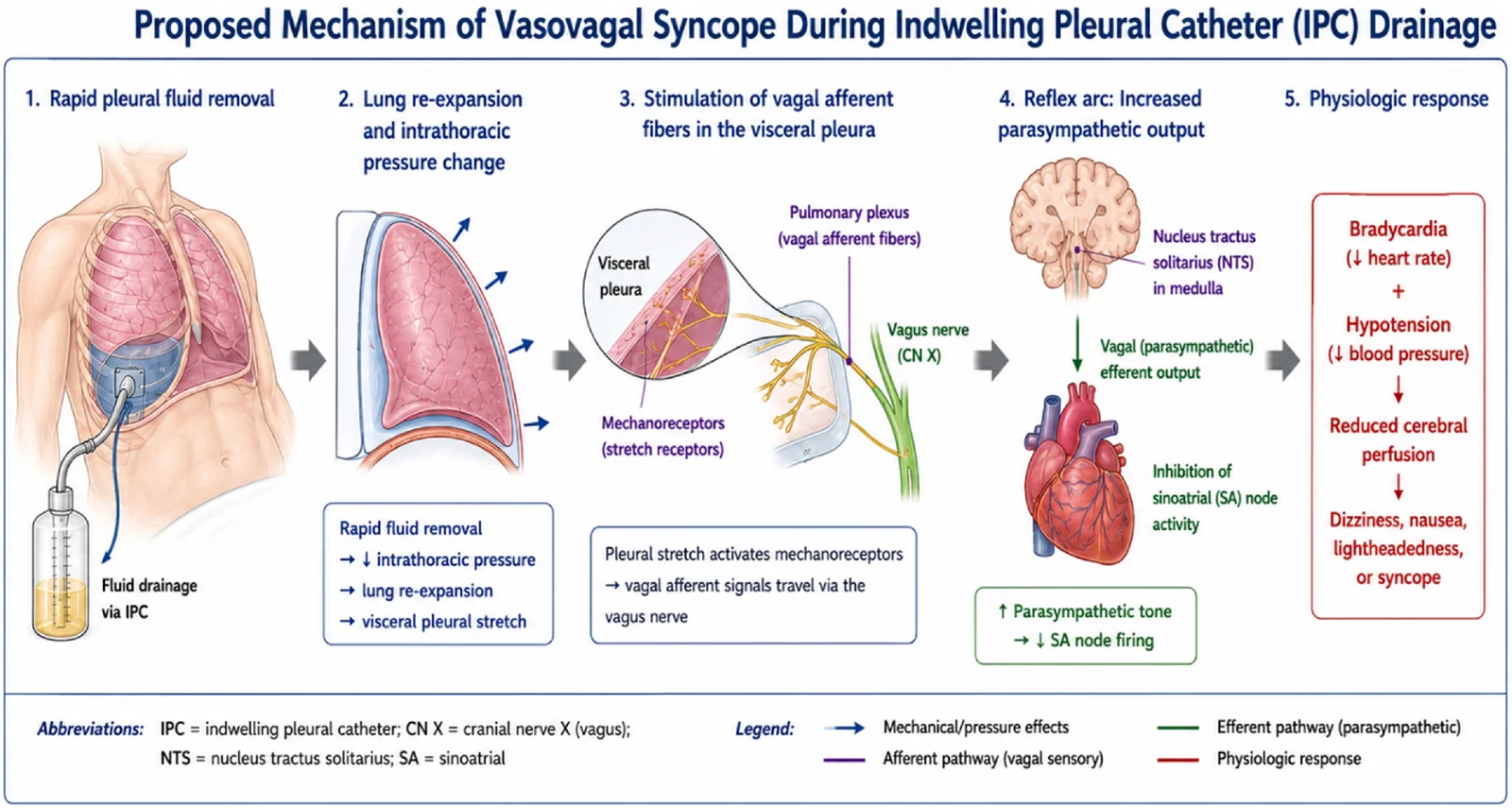
Mechanism of vasovagal syncope during indwelling pleural catheter (IPC) drai.

Vagally mediated bradycardic events have been reported in other thoracic interventions, including pleural irrigation, chest tube placement, and thoracostomy, supporting a shared reflex pathway [11], [12], [13]. However, the reproducibility of symptoms during routine IPC drainage in the outpatient setting suggests that this may represent an underrecognized and clinically relevant phenomenon. Of note, IPC databases classify non-infectious mechanical issues under ‘catheter malfunction’ or ‘pain’ meaning that there is likely underreporting of home-based hemodynamic events [14].

The observation that symptoms occurred toward the end of drainage suggests a threshold effect, whereby maximal lung re-expansion and pleural stretch produce the greatest autonomic response. Though systematic reviews show that thoracentesis can be performed safely up to 1.5 L without routine monitoring, individual hypersensitivity thresholds may vary [15]. Patient-specific factors, including age-related autonomic dysregulation, may further predispose exaggerated vagal responses and blunted baroreflex sensitivity [16], [17].

Additionally, the patient's use of atenolol likely exacerbated these episodes by blunting compensatory sympathetic responses. Discontinuation of beta-blockade is currently recommended for management of recurrent reflex syncope [18]. Additionally, slower drainage and supine positioning improve central venous pooling and stabilize cardiac preload, resulting in improved tolerance, highlighting the importance of both physiologic and iatrogenic contributors [19].

Importantly, alternative causes of syncope, including arrhythmia and structural heart disease, were excluded through repeated evaluation, strengthening the conclusion that these events were reflex-mediated and temporally linked to pleural drainage.

This case highlights a complication of pleural drainage that is not commonly reported and emphasizes the need for clinician awareness during such procedures.

**Clinical Implications**

● Procedural Modification: To reduce risk, drainage should be performed slowly with careful monitoring of vital signs, especially toward the end of the session.
● Early Cessation: The procedure should be stopped immediately if symptoms like dizziness occur.
● Patient Education: Patients must be taught to recognize early warning symptoms (lightheadedness, nausea) when performing home drainage.
● Medication Review: Clinicians should consider adjusting chronotropic medications, such as beta-blockers, in patients who demonstrate vagal hypersensitivity during drainage.
● At Home Care: Patients with recurrent vasovagal episodes and a lack of support at home should be counseled on available home healthcare options for assistance with drainage.

## CRediT authorship contribution statement

**Parita Shah:** Writing – review & editing, Writing – original draft, Data curation. **Lennon Colin:** Writing – review & editing, Data curation. **Irvin Lien:** Writing – review & editing, Validation, Supervision, Resources. **Pushan Jani:** Visualization, Supervision, Resources, Formal analysis, Data curation, Conceptualization.

## Financial disclosures

None.

## Declaration of competing interest

The authors declare that they have no known competing financial interests or personal relationships that could have appeared to influence the work reported in this paper.
